# Transplantation of Organs from Hepatitis C Virus-Positive Donors under Direct-Acting Antiviral Regimens

**DOI:** 10.3390/jcm11030770

**Published:** 2022-01-31

**Authors:** Muhammad Nauman Zahid

**Affiliations:** Department of Biology, College of Science, Sakhir Campus, University of Bahrain, Sakhir P.O. Box 32038, Bahrain; nzahid@uob.edu.bh; Tel.: +973-332-052-42

**Keywords:** liver transplantation, Hepatitis C virus, direct-acting antiviral agents, organ transplantation, Hepatitis C infection, HCV-positive donors

## Abstract

There is a discrepancy between the patients requiring organ transplants and the donors available to meet that demand. Many patients die every year while on the waiting list, and there is a need to bridge this gap. For many years, medical practitioners have been apprehensive of using donor organs from donors who have tested positive for the Hepatitis C virus (HCV), and with good reason. HCV has been proven to be among the leading causes of liver diseases requiring liver transplants. Over the years, studies have been carried out to find a treatment for Hepatitis C. The advent of direct-acting antivirals revolutionized the medical world. These medication regimens have been proven to treat Hepatitis C in transplant patients effectively. This systematic review will examine how DAA treatments affect transplants of different organs from HCV-positive donors to HCV-negative recipients.

## 1. Introduction

### 1.1. Overview

Globally, organ shortages are a big issue. Hepatitis C virus (HCV) affects 2–3% of the population worldwide [1]. The American Transplant Association estimates that almost 107,000 Americans await a lifesaving graft transplant. The overall number of deceased donors increased from 11,870 in 2019 to 12,588 in 2020 [2]. Homicides, suicides, and child abuse deaths all increased in 2020 compared to 2019. Despite the increase in deceased donors, 17 people die every day due to a dearth of available organ donors [2].

A deceased donor’s gift of organ donation can save up to eight lives, and improve many more [2]. Organ recipients are chosen based on need, donor compatibility, and proximity. Since 1988, 850,000 organs have been transplanted. Regardless, the organ transplant waiting list continues to increase. As such, we need to find other ways to get patients off the transplant list and into healthy, rewarding lives. The paucity of organ donors is global. According to UK statistics, 7000 people require organ transplants. In 2020, 470 people died while waiting for a qualified donor [3].

Most people with acute HCV are asymptomatic, but between 55 and 85% develop chronic HCV, with a 15 to 45 percent chance of developing liver cirrhosis within 20 to 30 years [1]. Cirrhosis puts people at risk of fatal liver disease. Hepatitis C continues to be the major cause of liver transplants. 

### 1.2. Hepatitis C Virus

Hepatitis C is a liver disease caused by a blood virus known as Hepatitis C [4]. According to the CDC, most infections in the US are caused by sharing needles and other injecting equipment [5]. Infection with HCV causes only a mild illness in some persons. However, it causes a long-term sickness that kills almost half of those affected [6]. Hepatitis C can cause liver cirrhosis, malignancy, and even death. Infected persons may not be aware they have it because they are not unwell enough to be hospitalized [2]. The disease has no vaccination, although well-tolerated curative treatments have been discovered over time [7]. The easiest way to avoid getting Hepatitis C is to avoid activities such as sharing needles.

### 1.3. Effect of Hepatitis C on Patients in Solid Organ Transplants

The prevalence of HCV infection among transplant patients varies by organ category and by regional epidemiology [8]. Patients on hemodialysis or with terminal renal disease are at greater risk. The frequency in dialysis patients directly relates to the period of dialysis and the nature of dialysis, and the quantity of blood and blood products transfused [8]. Over the last two decades, the incidence of Hepatitis C virus in dialysis patients has decreased due to increased blood screening and strict control methods [9]. Immunosuppression following kidney transplantation has been linked to early-onset and greater cirrhosis and its consequences in HCV patients [10,11]. Although HCV appears to not affect overall kidney transplant patient survival, fibrosing cholestatic hepatitis and liver failure cause 1–2% of early post-transplant fatalities [11,12,13]. On average, HCV-infected people live ten years less than non-infected patients, with liver failure and sepsis being the primary causes [10]. HCV sufferers are more likely to develop diabetes, chronic rejection, viral complications, and other health issues [10]. HCV-positive organs were only used in patients with life-threatening conditions or previous HCV infections [14]. Recent research shows that receiving a liver from an HCV-positive donor increases the risk of HCV transmission by 16% [15]. Scientists have discovered strategies to limit the diversity of Hepatitis C positivity on recipient patients to boost the efficiency of HCV-positive donor transplants (DAAs).

Using HCV-positive donor organs in HCV-negative patients has both benefits and drawbacks. As previously stated, there is a huge shortage of organs, with more individuals needing transplants every day. Using HCV-positive organs can minimize mortality and patient wait times. Similarly, direct-acting antiviral agents (DAA) have a high cure rate [14]. However, utilizing HCV-positive patients has drawbacks. The risk of 100% HCV transfer to the recipient is high, negatively impacting their quality of life. DAA therapy is also restricted in availability, and is expensive [14]. This review discusses data on HCV-positive donors and HCV-negative recipients receiving organ transplants, as well as DAA therapy outcomes.

## 2. Materials and Methods

### 2.1. Search Strategy

The systematic review used for this analysis adopted the recommendations of Moher et al. [16]. A systematic literature analysis was carried out using the Cochrane Library, PubMed, Embase, PubMed Central, Medline, Science Direct, and Google Scholar, revealing a database of studies on the transplantation of organs from Hepatitis C virus-positive donors under direct-acting antiviral regimens as of 30 September 2021. The study period was narrowed to the period starting from January 2011 to cover an era when DAA therapy was used. The search strategy was based on identifying keywords, for example, ‘Hepatitis C virus’ or ‘HCV’; ‘Hepatitis C Virus positive’; ‘HCV positive organ donor’; ‘HCV negative recipient’; ‘liver transplant’; ‘heart transplant’; ‘kidney transplant’; ‘direct-acting antivirals’; ‘DAA therapy.’

### 2.2. Study Identification

All academic material, ranging from clinical trials to cohort studies, article and book reviews, case study reports, or forum summaries, were considered if they documented at least one patient who was treated using an interferon-free Hepatitis C treatment procedure after organ transplantation. After eliminating replicas by the association of DOI, all abstracts from isolated documents were scrutinized for aptness.

The benchmarks used for exclusion were based on studies published on pediatric patients (below the age of 18 when they underwent treatment and transplantation), and those with inadequate or inconclusive data. References in the selected documents were then examined to find additional material for consideration.

## 3. Results

**Figure 1:**
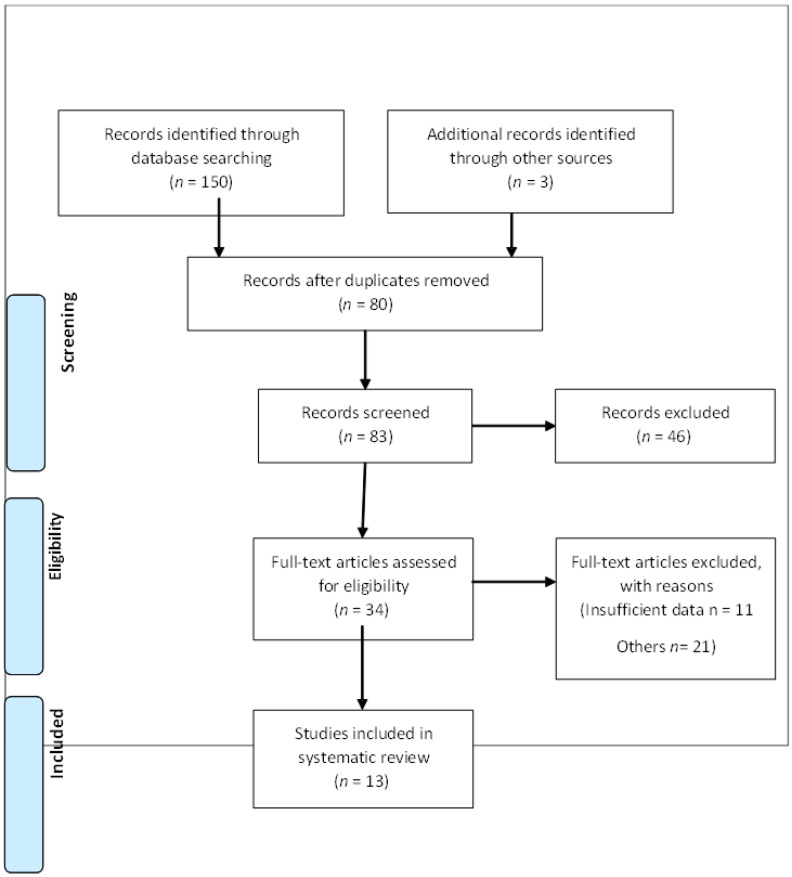
Process of searching, retrieving and selecting potentially relevant studies.

An internet search of the keywords revealed 150 publications. The duplicates were removed, and 80 publications remained. The remaining publications were screened, and 34 of them remained to be applied. After reading the articles, 13 articles were viable. The PRISMA flow diagram below illustrates the elimination of publications (Figure 1). Some studies did not provide sufficient information for the review, but they offered adequate context to the subject matter. Table 1 shows a summary of the findings.

Northup et al. looked at HCV-positive liver grafts [17]. They observed the US Organ Procurement and Transplantation Network’s Scientific Registry from 1994 to 2008, and found 56,275 liver transplants, over 19,496 HCV+ patients, and 934 HCV+ donors. Patient and graft survival were determined by the anti-HCV donor and recipient status. When an anti-HCV negative (HCV) recipient/HCV+ donor was used as the reference, the adjusted hazard ratio for mortality was identical for HCV-positive recipient/HCV-negative donors (1.176 vs. 1.165, *p* = 0.91). However, careful donor and recipient screening is required to maximize the usage of these extended criterion donors.

Ballarin et al. compared 63 patients who received anti-HCV-positive donor grafts to 63 patients who received anti-HCV-negative donor grafts [18]. A 5-year cumulative survival rate of 83.6 percent for patients who received anti-HCV positive grafts compared to 95.1 percent for the control group. Overall patient and graft survival did not differ between the two groups (*p* = 0.22 and 0.11). Hepatitis C recurrence was faster in those who received anti-HCV grafts, but not statistically significant (*p* = 0.07).

Patients with primary or secondary liver transplants were investigated by Charlton et al. [19]. They provided all patients 400 mg sofosbuvir twice a day for 24 weeks, with ribavirin 400 mg every other day, as needed. After 12 weeks of treatment, there was a consistent virologic response. Patients were mostly white, with an HCV genotype 1 infection rate of 83%, 40% cirrhosis (based on biopsy), and 88% prior interferon treatment. After 12 weeks, 28 of 40 patients exhibited a persistent virologic response. Relapse caused all virologic failures. No patients developed virus resistance during or after therapy. Symptoms included fatigue, diarrhea, anemia, and headache. An interferon-free treatment for HCV infection after transplantation is sofosbuvir plus ribavirin for 24 weeks, they concluded.

Berenguer et al. studied 611 patients in 19 research papers [20]. PEG-IFN alfa-2b was used in 16 studies [20]. The average SVR was 30.2% (range, 8–50 percent). There was a lot of dosing reduction and therapy cessation (73 percent and 27.6 percent, respectively). Treatment discontinuation and dose reductions due to side effects were widespread, and may have hampered SVR. Pungpapong et al. followed 123 patients on simeprevir and sofosbuvir for 30 weeks on average [21]. SVR12 was achieved in 90% of patients. At therapy week 4, half of the patients had undetectable HCV RNA, and their SVR12 rate was 96% (83 percent). A 12-week all-oral interferon-free antiviral regimen of simeprevir and sofosbuvir with or without RBV was well tolerated, and resulted in excellent SVR12 rates.

In another study, Forns et al. used sofosbuvir (SOF) and ribavirin (RBV) to treat patients with severe recurrent Hepatitis C, including those with fibrosing cholestatic hepatitis (FCH) and decompensated cirrhosis, who had a life expectancy of one year or less [22]. They provided SOF plus RBV to patients for 24–48 weeks. The researchers added pegylated interferon at their discretion. The data from the first 104 patients showed that 52 patients had an early severe recurrence (found 12 months after LT), and 52 had cirrhosis (diagnosed >12 months after LT). SVR was achieved in 54 (59%) of 92 patients assessed 12 weeks after treatment completion, with a greater prevalence (73%) in patients with early severe recurrence (35 of 48). Severe adverse events occurred in 49 patients, with hepatic decompensation being the most common. SOF and RBV exhibited significant SVR rates in individuals with severe recurrent HCV, including those with FCH and cirrhosis [22].

To assess the influence of antiviral medicines on HCV recurrence following liver transplantation, Leroy et al. studied 23 patients with FCH who took part in a cohort study in France and Belgium. The median time between transplantation and treatment was 5 months [23]. They treated 15 people with sofosbuvir plus daclatasvir, and 8 people with sofosbuvir and ribavirin for 24 weeks. All patients lived until week 36 without requiring re-transplantation. Sofosbuvir therapy with daclatasvir or ribavirin led to considerable clinical improvement and high virologic response rates at week 12.

Kapila et al. studied 77 HCV-negative recipients of solid organ transplants from HCV-positive donors [24]. None of the individuals had advanced hepatic fibrosis. DAA medication was started or completed in 58 patients who received kidney transplants (KT). Forty-one were able to attain SVR12, ten had undetectable viral loads but were not eligible for SVR12, and seven were still receiving treatment. One KT patient did not respond due to non-structural protein 5A resistance. Four patients received a liver transplant, and two received a liver-kidney transplant. Three patients achieved SVR12, one completed DAA therapy, and two remain on treatment. They found that using HCV-viremic grafts in the DAA era is safe and effective in carefully chosen individuals.

Durand et al. investigated the kidney transplantation from deceased donors aged 13 to 50 who had HCV RNA and antibodies [25]. Before transplantation, all recipients received 100 mg grazoprevir and 50 mg elbasvir. They continued to receive GZR-EBR for 12 weeks following transplantation for donors with genotype 1 infection, whereas those with genotype 2 or 3 infections received sofosbuvir 400 mg in addition to GZR-EBR for a total of 12 weeks of triple therapy. Twelve weeks after treatment, none of the 10 HCV-positive donor (D+)/negative recipient (R−) transplant patients had HCV RNA in their blood. The HCV treatment was safe and effective in preventing chronic HCV infection in HCV D+/R− patients.

Between September 2016 and March 2017, Schlendorf et al. studied 12 HCV-naive patients and 1 HCV-treated patient who received hearts from HCV-positive donors [26]. HCV patients were treated with DAAs. Nine of thirteen patients developed HCV viremia after transplant, including eight who finished DAA and were cured. All patients who received DAAs tolerated them well. Using HCV-positive donors could be a safe way to extend the donor pool in an era of highly successful DAAs. Long-term effects will require more research.

## 4. Discussion

### 4.1. Liver Transplantation

More than 100,000 people in the United States are awaiting a life-saving graft transplant, according to the American Transplant Association. In 2020, the overall number of dead donors increased from 11,870 to 12,588 in 2020 [2]. This is caused by various factors, especially opioid usage increasing from deceased donors. The transplant waiting list expands by one name every nine minutes, and despite an increase in the number of deceased donors, 17 individuals die every day due to a dearth of eligible organ donors [2]. A donor can be a living or deceased person, but the organ recipients are chosen primarily based on the need and the compatibility with the donor.

Initially, there was low enthusiasm towards using HCV-positive donors for liver transplants based on concerns about reduced organ viability, and the danger of severe viral hepatitis, among other challenges. Regardless, the number of HCV-negative donors continued to be abysmal [27]. In recent studies before the development of DAA treatment, it was demonstrated that the use of HCV-positive donors with HCV-positive patients offered similar organ efficiency and patient endurance to the use of HCV-negative livers in liver transplants. The most important study to recount the outcome of HCV-positive donor livers in HCV-positive liver transplant patients analyzed the U.S. Organ Procurement and Transplantation Network Scientific Registry statistics [17]. The study verified that there was no variance in the general endurance of patients amongst those who received HCV-positive donor livers and those who received the HCV-damaging organs. The difference in proportion between the two groups was clinically negligible, with HCV-positive donors having 1.82, and HCV-negative donors having 1.78. Ballarin et al., in a multicenter European case study, established that there was no variance in patients’ life expectancy between HCV-positive and HCV-negative donors [18]. These studies suggest that there are no differences in the survival amongst viremic and aviremic HCV-positive grafts. They also imply that there is no dissimilarity between HCV-positive and HCV-negative donors [27].

### 4.2. DAA Medication for HCV-Positive Liver Transplant Recipients

HCV positivity is defined as a positive HCV antibody (Ab) in a donor. A donor’s high-risk behavioral features may put them at risk of spreading HCV due to the window period, or the interval between infection and detection by a certain testing procedure [16]. A nucleic acid testing (NAT) assay that detects HCV virus RNA in the donor’s blood is advised for these high-risk donors. NAT tests reduced the time between HCV infection and detection from 70 days to 3–5 days. The advent of NAT has led to a redefinition of the phrase “HCV-positive donor” by the American Society of Transplantation (ASTS) [28]. A NAT-negative (nonviremic) HCV donor indicates that the infection has cleared up on its own or has been successfully treated. An HCV-seropositive and NAT-positive donor (viremic) indicates active infection and a high risk of disease transmission. HCV transmission is highly permissive in recipients of HCV-positive donor organs, with infection based on several genetically distinct donor viruses. However, at week 12 after medication, all participants achieved clinical cure from HCV infection with SVR, and have maintained HCV clearance [29].

HCV-positive liver transplant patients with active infections stand a risk of HCV recurrence connected with poorer post-HCV endurance compared to patients who initially did not have HCV [30]. Until recently, the treatments for recurrent HCV were limited by the unavailability of pharmaceutical therapy. Treatments with pegylated-interferon and ribavirin were badly endured in Hepatitis-C-positive patients undergoing a liver transplant. A systematic review involving 19 studies revealed that the average sustained virological response (SVR) in the patient population was only 30.2%, partly caused by adverse effects and treatment discontinuation [20]. Therefore, the usage of DAA medication revolutionized treatments of HCV infections in liver transplant patients [27].

Several studies have proved that the treatment is safe and efficient. In a prospective open-label study by Charlton et al., it was established that in 40 HCV patients with recurrent or compensated HCV infections, interventions with sofosbuvir and ribavirin resulted in sustained virological response at 12 weeks (SVR12) in 70% of the patients [19]. Another study involving 123 HCV-positive liver transplant patients that were treated with simeprevir and sofosbuvir with or without ribavirin achieved an SVR rate of 90%, with a more significant number of them suffering only minor aftermaths [21].

Studies have also been conducted on liver patients with more severe liver conditions. In a study involving 104 participants with a short life span and grievous liver diseases such as fibrosing cholestatic hepatitis and decomposing cirrhosis, the study revealed that among patients treated with sofosbuvir and ribavirin, whether or not pegylated-interferon was part of the treatment, 59% of them showed an SVR [22]. Charlton et al. also found, in a study involving 229 patients, that 93% of them achieved SVR 12. The efficacy of DAA treatment for fibrosing cholestatic hepatitis was shown in a survey by Leroy et al. in which 22 out of the 23 patients with fibrosing cholestatic hepatitis achieved SVR 12 after being treated with sofosbuvir and ribavirin with or without pegylated-interferon, or after being treated with sofosbuvir and daclatasvir with or without ribavirin [23]. The use of DAA medication in liver transplant recipients is promising for patients with recurrent HCV infections. Regardless, the efficacy of these interventions in recipients of HCV-positive livers is yet to be determined.

Clinical trials in liver and kidney transplant recipients have shown that DAAs have a high rate of cure. The SOLAR-1 and SOLAR-2 studies treated almost 400 liver transplant recipients with sofosbuvir/ledipasvir and weight-based ribavirin, with HCV cure rates ranging from 93 to 96 percent [31]. Colombo et al. randomly allocated 114 adult patients with an eGFR of less than 40 mL/min/1.73 m^2^ to either 12 or 24 weeks of sofosbuvir-ledipasvir 400 mg/90 mg combination therapy; 100% of the patients were cured [32]. A single-arm trial of 10 patients received orthotopic heart transplantation (OHT) from HCV-infected donors into uninfected recipients, followed by elbasvir/grazoprevir treatment [33]. HCV RNA levels in the recipients ranged from 25 to 40 million IU/mL, but after treatment with elbasvir/grazoprevir, HCV NAT levels in all 10 patients dropped dramatically. Nine people experienced a virologic response that lasted longer than 12 weeks (SVR-12) [33].

HCV grafts with no or mild fibrosis should be considered until more trials with histological assessment of grafts following DAA treatment are available. There are currently no published criteria for selecting HCV-positive donors based on age and hepatic steatosis. It has been reported that older donors (>50 years) had more advanced fibrosis than younger donors (50 years) [34]. Similarly, Lai et al. reported a connection between HCV status and progressive fibrosis only in donors 45 years old [34]. The impact of donor steatosis on liver transplant outcomes varies by the study because most transplant surgeons reject these organs, and no data on using HCV-positive fatty grafts are currently available.

Transplanting viremic donors into non-viremic HCV recipients raises ethical concerns. “Should hepatitis C antibodies be used in transplantation?” is a question that was asked in a 1995 Lancet editorial [35]. HCV transmission into negative donors presents ethical considerations that are already a part of the standard of care in transplant therapy. The most important components of autonomy in HCV donor positive/recipient negative (D+/R) liver transplant are informed consent and institutional precautions for drugs that are not yet the standard for treatment (LT). For patients to make autonomous decisions regarding their care, standardized informed consent would be required. There is a need for rigorous, well-designed randomized controlled trials (RCTs) to examine the outcomes of HCV D+/R LT.

The growing disparity between donor organs and transplant recipients has made it an ethical imperative for transplant specialists to discover strategies to enhance transplant access. The increased mortality of patients on the waiting list gives a compelling rationale for using HCV viremic organs in nonviremic recipients. Large clinical trials are needed to prove the procedure’s safety. Despite this, HCV transmission to transplant recipients is still a big concern. Surgical difficulties, post-transplant immunosuppression, and mortality are now generally recognized risks of organ transplantation [35].

### 4.3. Transplantation of Other Organs

Within the context of transplantation, DAA appears to be safe and has proved effective and safe in HCV treatment. Even in heterogeneous studies, there are no reported relapses after a complete DAA therapy. In the THINKER that was declared on kidney transplants from viremic HCV donors to HCV-negative recipients, it was found that one patient had a high HCV viral load upon follow-up measurements during therapy on account of DAA resistance, and therefore necessitated a change in regimen [36]. In Cleveland Clinic, Florida, the experience with kidney transplants of HCV-positive organs was successful except for only one treatment, with 41 patients achieving SVR 12 [24]. Another EXPANDER study observed kidney transplant recipients not restricted to genotype 1. All patients received 12 weeks of elbasvir-grazoprevir with the first dose on call to the operating room, and a median time from the first dose to reperfusion of the transplanted kidney of 5.1 h. For three out of the ten patients who had genotype 2 or 3, sofosbuvir was added, and all the patients achieved SVR 12 [25].

In the interferon era, the experience with heart donors was disheartening, laden with the development of coronary vasculopathy, progressive liver disease, and lower chances of survival [37]. However, HCV-positive organs have shown positive results in the DAA era. Researchers in Vanderbilt University Medical Centre (VUMC) reported an early study with 11 HCV-positive donors into HCV-negative recipients [26]: treatment was started 19 to 48 days after the operation using sofosbuvir-ledipasvir, or sofosbuvir-velpatasvir with SVR12 in 8 of 9 [38]. The ninth patient succumbed to pulmonary embolism before achieving SVR12. The authors further reported an updated experience in April 2019 with 70 HCV-positive donors. HCV viremia developed in 67 of 70 (96%), and all 37 who completed treatment achieved SVR12 [26]. Other studies have reported similar outcomes after heart and lung transplants of HCV-positive organs.

### 4.4. Transplantation of Hepatitis-C-Positive Organs under Direct-Acting Antiviral Treatment

From the above analysis, it is apparent that DAA treatment effectively manages the adversities of the Hepatitis C virus in transplant patients. Before DAA treatment was invented, patients experienced reduced graft and patient survival. After DAA was introduced, studies showed that transplanted grafts’ short-term survival improved compared to those treated before DAA treatment was invented [39]. Also, recently, in an analysis of the European Liver Transplant Registry, it was found that there was an improved survival rate in liver transplant patients with HCV-related liver problems when DAA treatments were used [40]. However, little is known about the long-term implications of DAA treatment in liver transplantation patients [41]. Usually, the risk of a transplant patient getting Hepatitis C from positive graft donors depends on how much of the virus the donor organ has [42]. Some donors are HCV-positive, but they have a negative viral load. In that case, there is a shallow risk of HCV being transmitted to the recipient, even for liver transplant patients [14,15,28]. Therefore, there is a need to monitor recipients of organs from high-risk donors [43]. Kapila et al. found that the risk of transmission from high-risk donors was approximately 100% in liver transplant patients. In contrast, transplant patients of other organs saw lower levels of information of Hepatitis C [24]. Regardless of these statistics, when used with DAA treatment regimens, the recipients showed impressive outcomes. Out of the 77 patients, 76 reached sustained virologic response [24]. It has been proven empirically, and on many occasions, that DAA treatments are effective in treating the Hepatitis C virus in patients with liver diseases, chronic kidney diseases, and heart diseases.

With the discussion above, it is now possible for patients who are HCV-negative to receive grafts from donors with Hepatitis C virus with proper management before transplantation, and adequate administration of DAA treatment upon transplantation [44]. In applying DAA treatment regimens to improve the viability of HCV-positive organs, there is usually no time to carry out HCV genotyping because of the time pressure associated with organ transplants, and the logistical technicalities [44]. To ease this process, pan-genotypic measures are employed, which will cover all HCV-positive organs [44]. Several regimens have been approved for use over the years. The combination of sofosbuvir and velapatasvir has proved to be as effective as the combination of sofosbuvir and ledipasvir, with the benefit of treating all genotypes. As it stands, it is the recommended treatment for patients who are not getting transplants with close to perfect outcomes [44]. In 2017, the treatment regimen of glecaprevir and pibrentasvir was the only treatment that had been approved for use with patients who have severe kidney failure or who are undergoing dialysis [45].

It is essential to identify the effective regimens for different patients, and educate patients and the public on the effectiveness of DAA treatments in dealing with the Hepatitis C virus. The recipients considered for the transplantation with HCV-positive organs accompanied by DAA treatments should be those who can stick to the DAA therapy unrelentingly [45]. Similarly, patients with a high risk of developing complications, or in whom the disease may return and interfere with the implementation of the DAA treatment, must not be included in the study. When educated on the revolutionary changes in medicine allowing Hepatitis C virus-positive donor organs in organ donors, patients and their families can use HCV-positive organs under DAA treatment with an assurance of its effectiveness.

### 4.5. Limitations of the Study

The study has shown several limitations in that it has not addressed how some factors affect the effectiveness of DAA intervention. Additionally, given the types of studies included in this review, there is a possibility of finding a positive outcome to the research. This review does not also address the safety of DAA in organ transplants concerning other factors. It has also failed to address the effectiveness of DAA treatment regimens in patients requiring transplantation of other organs aside from the heart, kidney, and liver.

## 5. Conclusions

It is a fact that many patients around the world require organ transplants, yet the donor list does not grow to meet the need. There is a need to address the shortage. The Hepatitis C virus has been a reason for medical practitioners to shun the use of organs. Many deceased donors could contribute towards the solution, except for the fact that they have Hepatitis C, which is among the leading causes of liver complications. Scientists, however, over the years, have developed treatments that can be used to treat the Hepatitis C virus, therefore making HCV-positive organs viable for transplantation. In the past, interferon interventions that were used to counter the effects of the disease proved ineffective and largely unreliable, leaving transplant recipients open to chronic liver problems and death. With the invention of direct-acting antiviral treatments, the recovery rate of HCV-positive patients has improved. Statistics have shown that the patients who have received HCV-positive organs under DAA treatment regimens have achieved sustainable virological response within 12 weeks. This is encouraging, since it shows that with suitable therapy, treatment, and monitoring, patients can receive organs from Hepatitis C-positive donors, and proceed to live fulfilled lives when DAA treatment regimens are followed.

This study set out to determine how DAA treatments affect transplantation of Hepatitis-C-positive organs to both HCV-positive and negative recipients. We have found that DAA treatments have seen more significant successes than treatments used in the past. There is, however, a need to make sure that the treatments become effective, requiring the patients to adhere to scrupulous care to ensure efficiency. Regardless, the confidence in the effectiveness of DAA regimens continues to soar as research into the matter continues to result in more precise regimens to address all scenarios.

## Figures and Tables

**Table 1:** Characteristics of the included studies.

S.No	Author	Year	Study Design	Population Study	Outcome
1.	Northup PG	2010	Multivariable Analysis	934	The study found no variance in the total number of patients that survived between those who were given HCV-positive donor livers and those who were given the HCV-negative. The relation index between the two clusters was clinically inconsequential at 1.82 for HCV-positive donors versus 1.78 for HCV-negative donors.
2.	Ballarin et al.	2011	Case-Control Study	63	This study also showed no variation in the number of patients who survived at 1 and 5 years (*p* = 0.22 and 0.11, respectively). Allograft pathology revealed that HCV-positive donor livers had higher histological activity index numbers (17.5% vs. 3.2% for scores 5–8, *p* = 0.01) and stage I fibrosis (52.4% vs. 17.5%, *p* < 0.001).
3.	Berenguer M et al.	2008	Systematic Review	19 studies, 611 patients	The mean SVR in this patient cohort was only 30.2%, partly due to unfavorable events that required doses to be reduced or the treatment to be terminated.
4.	Charlton et al.	2015	Prospective Open-Label Study	40 patients	Interventions with sofosbuvir and ribavirin occasioned a sustained virological response at 12 weeks (SVR12) in 70% of the patients.
5.	Pungpapong S et al.	2015	Multicenter Study	123 HCV-positive liver transplant patients	In the subjects that received interventions with simeprevir and sofosbuvir with or without ribavirin, 90% achieved SVR, with the bulk of patients suffering only slight adverse repercussions.
6.	Forns et al.	2015	Case Study	104 patients	This study established that treatment with sofosbuvir and ribavirin, with or without pegylated intervention, resulted in SVR 12 in 59% of the patients.
7.	Leroy et al.	2015	Case Study	23 patients	22 of 23 patients (96% of the population) with fibrosing cholestatic hepatitis achieved SVR12 following treatment with sofosbuvir and ribavirin with or without pegylated-interferon or sofosbuvir and daclatasvir with or without ribavirin.
8.	Kapila N et al.	2020	Case Study	45 patients	Cleveland Clinic Florida reported their findings on kidney transplantation of HCV-positive organs, with only one of the treatments failing: 41 patients achieving SVR12.
9.	Durand CM et al.	2018	Case Study	Ten patients	Their EXPANDER study registered ten kidney transplant recipients, but it did not restrict them to genotype 1. Determining the HCV genotype was done at the time of transplantation, and reported in a week. All the recipients were given 12 weeks of elbasvir-grazoprevir. The initial dose was given on call to the operating room and midway through from the initial amount to reperfusion of the new kidney at 5.1 h. For 3 out of the 10 patients who had genotype 2 or 3, sofosbuvir was included in the regimen. All the patients attained SVR12.
10.	Schlendorf KH et al.	2018	Case Study	37 patients, heart transplant	In this study, all 37 patients who took the treatment to completion achieved SVR12.

## Data Availability

Not applicable.

## References

[1] World Health Organization (2015). Hepatitis C: Fact Sheet NO164. http://www.who.int/mediacentre/factsheets/fs164/en/.

[2] American Transplant Foundation Facts and Myths about Transplant. https://www.americantransplantfoundation.org/about-transplant/facts-and-myths/.

[3] Organ Donation and Transplantation NHS Blood and Transplant. https://www.nhsbt.nhs.uk/what-we-do/transplantation-services/organ-donation-and-transplantation/.

[4] Centres for Disease Control and Prevention (2019). Viral Hepatitis Surveillance Report—United States. https://www.cdc.gov/hepatitis/statistics/2019surveillance/index.htm.

[5] Zibbell J.E., Asher A.K., Patel R.C., Kupronis B., Iqbal K., Ward J.W., Holtzman D. (2018). Increases in acute Hepatitis C virus infection related to a growing opioid epidemic and associated injection drug use, United States, 2004 to 2014. Am. J. Public. Health.

[6] Centres for Disease Control and Prevention (2012). Notes from the field: Hepatitis C virus infections among young adults—Rural wisconsin, 2010. MMWR Morb. Mortal. Wkly. Rep..

[7] Seifert L.L., Perumpail R.B., Ahmed A. (2015). Update on Hepatitis C: Direct-acting antivirals. World J. Hepatol..

[8] Belga S., Doucette K.E. (2016). Hepatitis C in non-hepatic solid organ transplant candidates and recipients: A new horizon. World J. Gastroenterol..

[9] Espinosa M., Martn-Malo A., Ojeda R., Santamara R., Soriano S., Aguera M., Aljama P. (2004). Marked reduction in the prevalence of Hepatitis C virus infection in hemodialysis patients: Causes and consequences. Am. J. Kidney Dis..

[10] Mathurin P., Mouquet C., Poynard T., Sylla C., Benalia H., Fretz C., Thibault V., Cadranel J.F., Bernard B., Opolon P. (1999). Impact of hepatitis B and C virus on kidney transplantation outcome. Hepatology.

[11] Zylberberg H., Carnot F., Mamzer M.F., Blancho G., Legendre C., Pol S. (1997). Hepatitis C virus-related fibrosing cholestatic hepatitis after renal transplantation. Transplantation.

[12] Delladetsima J.K., Boletis J.N., Makris F., Psichogiou M., Kostakis A., Hatzakis A. (1999). Fibrosing cholestatic hepatitis in renal transplant recipients with Hepatitis C virus infection. Liver Transpl. Surg..

[13] Toth C.M., Pascual M., Chung R.T., Graeme-Cook F., Dienstag J.L., Bhan A.K., Cosimi A.B. (1998). Hepatitis C virus-associated fibrosing cholestatic hepatitis after renal transplantation: Response to interferon-alpha therapy. Transplantation.

[14] Selzner N., Berenguer M. (2018). Should organs from Hepatitis C-positive donors be used in Hepatitis C-negative recipients for liver transplantation?. Liver Transplant..

[15] Bari K., Luckett K., Kaiser T., Diwan T., Cuffy M., Schoech M., Safdar K., Blackard J.T., Apewokin S., Paterno F. (2018). Hepatitis C transmission from seropositive, nonviremic donors to non-Hepatitis C liver transplant recipients. Hepatology.

[16] Moher D., Liberati A., Tetzlaff J., Altman D.G. (2009). Preferred reporting items for systematic reviews and meta-analyses: The PRISMA statement. PLoS Med..

[17] Northup P.G., Argo C.K., Nguyen D.T., McBride M.A., Kumer S.C., Schmitt T.M., Pruett T.L. (2010). Liver allografts from Hepatitis C positive donors can offer good outcomes in Hepatitis C positive recipients: A U.S. National Transplant Registry analysis. Transpl. Int..

[18] Ballarin R., Cucchetti A., Spaggiari M., Montalti R., Di Benedetto F., Nadalin S., Troisi R.I., Valmasoni M., Longo C., De Ruvo N. (2011). Long-term follow-up and outcome of liver transplantation from anti-Hepatitis C virus-positive donors: A European multicentric case-control study. Transplantation.

[19] Charlton M., Gane E., Manns M.P., Brown R.S., Curry M.P., Kwo P.Y., Fontana R.J., Gilroy R., Teperman L., Muir A.J. (2015). Sofosbuvir and ribavirin for treatment of compensated recurrent Hepatitis C virus infection after liver transplantation. Gastroenterology.

[20] Berenguer M.J. (2008). Systematic review of the treatment of established recurrent Hepatitis C with pegylated interferon in combination with ribavirin. J. Hepatol..

[21] Pungpapong S., Aqel B., Leise M., Werner K.T., Murphy J.L., Henry T.M., Ryland K., Chervenak A.E., Watt K.D., Vargas H.E. (2015). Multicentre experience using simeprevir and sofosbuvir with or without ribavirin to treat Hepatitis C genotype 1 after liver transplant. Hepatology.

[22] Forns X., Charlton M., Denning J., McHutchison J.G., Symonds W.T., Brainard D., Brandt-Sarif T., Chang P., Kivett V., Castells L. (2015). Sofosbuvir compassionate use program for patients with severe recurrent Hepatitis C after liver transplantation. Hepatology.

[23] Leroy V., Dumortier J., Coilly A., Sebagh M., Fougerou-Leurent C., Radenne S., Botta D., Durand F., Silvain C., Lebray P. (2015). Efficacy of Sofosbuvir and Daclatasvir in Patients with Fibrosing Cholestatic Hepatitis C After Liver Transplantation., Agence Nationale de Recherches sur le SIDA et les Hépatites Virales CO23 Compassionate Use of Protease Inhibitors in Viral C in Liver Transplantation Study Group. Clin. Gastroenterol. Hepatol..

[24] Kapila N., Menon K.V.N., Al-Khalloufi K., Vanatta J.M., Murgas C., Reino D., Ebaid S., Shaw J.J., Agrawal N., Rhazouani S. (2020). Hepatitis C virus NAT-positive solid organ allografts transplanted into Hepatitis C virus-negative recipients: A real-world experience. Hepatology.

[25] Durand C.M., Bowring M.G., Brown D.M., Chattergoon M.A., Massaccesi G., Bair N., Wesson R., Reyad A., Naqvi F.F., Ostrander D. (2018). Direct-acting antiviral prophylaxis in kidney transplantation from Hepatitis C virus-infected donors to noninfected recipients: An open-label nonrandomized trial. Ann. Int. Med..

[26] Schlendorf K.H., Zalawadiya S., Shah A.S., Wigger M., Chung C.Y., Smith S., Danter M., Choi C.W., Keebler M.E., MarshallBrinkley D. (2018). Early outcomes using Hepatitis C-positive donors for cardiac transplantation in the era of effective direct-acting antiviral therapies. J. Heart Lung Transplant..

[27] Bushyhead D., Goldberg D. (2017). Use of Hepatitis C-Positive Donor Livers in Liver Transplantation. Curr. Hepatol. Rep..

[28] Levitsky J., Formica R.N., Bloom R.D., Charlton M., Curry M., Friedewald J., Friedman J., Goldberg D., Hall S., Ison M. (2017). The American Society of Transplantation Consensus Conference on the Use of Hepatitis C Viremic Donors in Solid Organ Transplantation. Am. J. Transplant..

[29] Zahid M.N., Wang S., Learn G.H., Abt P.L., Blumberg E.A., Reese P.P., Goldberg D.S., Shaw G.M., Bar K.J. (2019). High multiplicity of infection following transplantation of hepatitis C virus–positive organs. J. Clin. Investig..

[30] Thuluvath P.J., Guidinger M.K., Fung J.J., Johnson L.B., Rayhill S.C., Pelletier S.J. (2010). Liver transplantation in the United States, 1999–2008. Am. J. Transplant..

[31] Charlton M., Everson G.T., Flamm S.L., Kumar P., Landis C., Brown R.S., Fried M.W., Terrault N.A., O’Leary J.G., Vargas H.E. (2015). Ledipasvir and sofosbuvir plus ribavirin for treatment of HCV infection in patients with advanced liver disease. Gastroenterology.

[32] Colombo M., Aghemo A., Liu H., Zhang J., Dvory-Sobol H., Hyland R., Yun C., Massetto B., Brainard D.M., McHutchison J.G. (2017). Treatment With Ledipasvir-Sofosbuvir for 12 or 24 Weeks in Kidney Transplant Recipients With Chronic Hepatitis C Virus Genotype 1 or 4 Infection: A Randomized Trial. Ann. Int. Med..

[33] McLean R.C., Reese P.P., Acker M., Atluri P., Bermudez C., Goldberg L.R., Abt P.L., Blumberg E.A., van Deerlin V.M., Reddy K.R. (2019). Transplanting hepatitis C virus–infected hearts into uninfected recipients: A single-arm trial. Am. J. Transplant..

[34] Khapra A.P., Agarwal K., Fiel M.I., Kontorinis N., Hossain S., Emre S., Schiano T.D. (2006). Impact of donor age on survival and fibrosis progression in patients with hepatitis C undergoing liver transplantation using HCV+ allografts. Liver Transplant..

[35] Couri T., Aronsohn A. (2019). When Theory Becomes Reality: Navigating the Ethics of Transplanting Hepatitis C Virus–Positive Livers Into Negative Recipients. Clin. Liver Dis..

[36] Bruno S., Nicole B., Dharan Nila J., Gail M., James N., Macdonald Peter S. (2019). Heart Transplantation From Hepatitis C–Positive Donors in the Era of Direct Acting Antiviral Therapy: A Comprehensive Literature Review. Transplant. Direct..

[37] Gasink L.B., Blumberg E.A., Localio A.R., Desai S.S., Israni A.K., Lautenbach E. (2006). Hepatitis C virus seropositivity in organ donors and survival in heart transplant recipients. JAMA.

[38] Kappus M.R., Wolfe C.R., Muir A.J. (2020). Direct-Acting Antivirals and Organ Transplantation: Is There Anything We Can’t Do?. J. Infect. Dis..

[39] Axelrod D.A., Schnitzler M.A., Alhamad T., Gordon F., Bloom R.D., Hess G.P., Xiao H., Nazzal M., Segev D.L., Dharnidharka V.R. (2018). The impact of direct-acting antiviral agents on liver and kidney transplant costs and outcomes. Am. J. Transplant..

[40] Belli L.S., Perricone G., Adam R., Cortesi P.A., Strazzabosco M., Facchetti R., Karam V., Salizzoni M., Andujar R.L., Fondevila C. (2018). Impact of DAAs on liver transplantation: Major effects on the evolution of indications and results. An ELITA study based on the ELTR registry. J. Hepatol..

[41] Cotter T.G., Paul S., Sandıkçı B., Couri T., Bodzin A.S., Little E.C., Sundaram V., Charlton M. (2019). Improved Graft Survival after Liver Transplantation for Recipients with Hepatitis C Virus in the Direct-Acting Antiviral Era. Liver Transpl..

[42] Dharani G., Nancy R. (2020). Liberalizing transplantation of HCV positive donor organs into HCV negative recipients. Dig. Med. Res..

[43] Suryaprasad A., Basavaraju S.V., Hocevar S.N., Theodoropoulos N., Zuckerman R.A., Hayden T., Forbi J.C., Pegues D., Levine M., Martin S.I. (2015). Transmission of Hepatitis C Virus From Organ Donors Despite Nucleic Acid Test Screening. Am. J. Transplant..

[44] Sise M.E., Strohbehn I.A., Bethea E., Gustafson J.L., Chung R.T. (2019). Balancing the risk and rewards of utilizing organs from Hepatitis C viremic donors. Curr. Opin. Organ. Transplant..

[45] Davis M.I., Chute D.F., Chung R.T., Sise M.E. (2018). When and how can nephrologists treat Hepatitis C virus infection in dialysis patients?. Semin. Dial..

